# Depth image conversion model based on CycleGAN for growing tomato truss identification

**DOI:** 10.1186/s13007-022-00911-0

**Published:** 2022-06-17

**Authors:** Dae-Hyun Jung, Cheoul Young Kim, Taek Sung Lee, Soo Hyun Park

**Affiliations:** 1 Smart Farm Research Center, Korea Institute of Science and Technology (KIST), Gangneung-si, Gangwon-do 25451 Republic of Korea; 2 Bit2Farm, Daehak 4-ro, Yeongtong-gu, Suwon-si, Gyeonggi-do 16226 Republic of Korea; 3 Graduate School of Mechanical Engineering, Yonsei University, 50, Yonsei-ro, Seodaemun-gu, Seoul, 03722 Republic of Korea

**Keywords:** Deep learning, Generative adversarial networks, Convolutional neural network, Robot platform

## Abstract

**Background:**

On tomato plants, the flowering truss is a group or cluster of smaller stems where flowers and fruit develop, while the growing truss is the most extended part of the stem. Because the state of the growing truss reacts sensitively to the surrounding environment, it is essential to control its growth in the early stages. With the recent development of information and artificial intelligence technology in agriculture, a previous study developed a real-time acquisition and evaluation method for images using robots. Furthermore, we used image processing to locate the growing truss to extract growth information. Among the different vision algorithms, the CycleGAN algorithm was used to generate and transform unpaired images using generated learning images. In this study, we developed a robot-based system for simultaneously acquiring RGB and depth images of the growing truss of the tomato plant.

**Results:**

The segmentation performance for approximately 35 samples was compared via false negative (FN) and false positive (FP) indicators. For the depth camera image, we obtained FN and FP values of 17.55 ± 3.01% and 17.76 ± 3.55%, respectively. For the CycleGAN algorithm, we obtained FN and FP values of 19.24 ± 1.45% and 18.24 ± 1.54%, respectively. When segmentation was performed via image processing through depth image and CycleGAN, the mean intersection over union (mIoU) was 63.56 ± 8.44% and 69.25 ± 4.42%, respectively, indicating that the CycleGAN algorithm can identify the desired growing truss of the tomato plant with high precision.

**Conclusions:**

The on-site possibility of the image extraction technique using CycleGAN was confirmed when the image scanning robot drove in a straight line through a tomato greenhouse. In the future, the proposed approach is expected to be used in vision technology to scan tomato growth indicators in greenhouses using an unmanned robot platform.

**Supplementary Information:**

The online version contains supplementary material available at 10.1186/s13007-022-00911-0.

## Background

In crops, the growing tip and roots where cell division occurs are sensitive to the surrounding environment. In particular, the hypertrophy of early reproductive growth of crops can be determined from the early stages of truss development [1], which can also affect the quality of flowers and fruits. Although experts can determine hypertrophy with the naked eye, this practice makes collecting accurate numerical data and setting crop management standards difficult. While studies are being actively conducted to analyze diseases using digital imaging on tomato crops, few have measured the indicators related to tomato growth. In the case of the growing truss of the tomato plant, it is difficult to collect numerical information from obtained images to determine a label value considering the lack of reference video images.

Developing a future-oriented agricultural robot platform is expected to reduce the challenges in acquiring image data comprising growth information [2] developed a mechanical robot arm with a high degree of freedom and an intelligent control unit that moves the arm by analyzing the captured images. Research on object recognition with a diversified view based on images using optimized robot arm placement is underway [3]. Chang et al. [4] reported the use of image processing techniques such as color space transformations, morphological operations, and 3D localization to identify objects and grippers in captured images, and estimate their relative positions using the computer vision area as a novel algorithm that identifies the object before determining the optimum movement of the robot arm. In agriculture, measuring growth using computer vision has been a research topic for a relatively long time [4, 5]. In particular, robots are used in harvesting, and various image processing techniques have been applied to harvest fruit and determine its ripeness [6–8]. Zhuang et al. [9] proposed a computer-vision-based method for locating acceptable picking points for litchi clusters, and image processing algorithm was used to track the location of the fruit while considering the agronomic characteristics of the picking point.

Although it is necessary to apply image processing techniques by identifying the characteristics of the crop, an image segmentation method has yet to be developed to distinguish the growing truss from other parts of the tomato plant. Because a tomato cultivation environment inside a greenhouse is dense, classifying stems or leaves using images is difficult [8, 10]. Thus far, researchers have not been able to distinguish tomato stems and leaves, the components of the growing truss, from other surrounding objects in RGB images. Xiang [11] performed crop segmentation using a simplified pulse coupled neural network by measuring 385 tomato images at night. The best results obtained from this segmentation technique were true and false rates of 59.22% and 13.77%, respectively. However, it could only be performed using a specific light at night for light correction, which requires more mechanical devices and technical improvements to measure the growing truss of the tomato plant. Zhang and Xu [12] reported a method for improving the accuracy of image segmentation in the middle and late stages of the fruit growth using an unsupervised method. However, this method could not differentiate tomato stems and leaves from other surrounding objects in RGB images. Many studies using RGB images have been used to target the tomato fruit, and many have reported the possibility of using these identification methods in tomato cultivation. However, segmentation studies on the growing truss of tomato plants have yet to be successfully reported.

To solve this problem, there is potential in the use of a 3D camera capable of segmentation according to distance or image processing techniques that are affected by solar light in greenhouses, and 3D-depth cameras are widely used in image acquisition platforms for recognizing objects in various industries, including agriculture [13–15]. It has been reported that a technology that combines depth and color image information recorded with a stereo camera (a 3D camera technology) can be used to classify objects [16, 17]. Unlike conventional 2D cameras, 3D-depth cameras can be distributed to the field and used to calculate the depth value of each pixel in an image, and research on growth measurement using 3D cameras is underway.

Deep learning image processing technology has advanced in recent years. For instance, in image recognition and classification, studies using convolutional neural networks (CNN) have been effectively applied to various industrial fields [18–21]. For example, Afonso et al. [22] used Mask-RCNN, which recognizes objects at high speed and is specialized for segmentation, for tomato fruit recognition and confirmed its potential in greenhouse environments. The structure of such a CNN has the form of general supervised learning, which requires the region of interest (ROI) in all image data to be annotated, and the accuracy of the model is dependent on some extent by the quantity and quality of the data obtained. Therefore, it is important not only to develop a robot platform to extract accurate images in an automated greenhouse, but also to apply an algorithm capable of self-learning with an appropriate number of images.

Generative advertising networks (GANs) have particularly gained wide attention [23, 24]. The basic GAN configuration comprises a deep learning technology that simultaneously learns the delimiter and generator model to obtain the target image from the generator, showing endless possibilities in unsupervised learning. GAN technology specifically aims to map input and output images using an image dataset called image to image translation. It is possible to color a black and white image, turn a day photo into a night photo, or make a border-only photo look like a real object. It is a technique frequently used in applications such as artificial intelligence coloring, photo restoration, and image transformation. A Pix-2-Pix and conditional GAN algorithm have been reported to convert paired images [25], which require paired image data of the same format. However, because a relatively large amount of training data is required, the CycleGAN algorithm has been proposed as an alternative to the traditional GAN as it has been trained to avoid switching between images through the learning of two unpaired images by circulating the two generators and identifiers [26, 27]. A representative application example of the CycleGAN is a study wherein an image of a zebra was converted to that of an ordinary horse. Researchers have reported [28] that this technology can switch the patterns of two images, such as a photo with depth information and a general image with RGB information. Furthermore, unlike other CNN algorithms, CycleGAN is a learning process that generates images by self-learning and requires a relatively small number of labeled image data. Therefore, the CycleGAN is expected to enable efficient algorithm application in environments where data acquisition is difficult, such as in a greenhouse.

Considering these points, the current research lacks detection technology for determining the growing truss of the tomato plant, and is therefore the goal of this study. For image acquisition using an unmanned robot, extraction of the growing truss must be performed on-site, requiring a segmentation technique that uses depth image information. The specific objectives of this study are:

1. Building a robot monitoring system that can identify a growing truss image.
2. Converting between RGB and depth images of greenhouse plants using the CycleGAN algorithm.
3. Verifying the application of a robot vision-based image separation technique to identify the growing truss of the tomato plant in a greenhouse.

## Methods

### Greenhouse environment and image acquisition device

The experiment was conducted in a greenhouse facility where tomatoes are grown. A 2000 m^2^ interlocking Venlo greenhouse was utilized, wherein the insides comprised of sensors and control systems to manage the level of carbon dioxide at a constant temperature and humidity. The location of the greenhouse is at latitude 37.7986 and longitude 128.8575. We used Dafnis tomatoes for the experiment, and images of the harvested tomatoes were collected approximately 180 days after planting. Tomatoes are grown in a greenhouse drip irrigation-based hydroponics system, and the nutrient solution is supplied through a solar proportional irrigation control. The roots of the tomatoes are established in a rock-wool substrate, and the substrate and the gutter supporting it are located at a height of about 1.3 m from the ground. The growing truss of the tomato plant is located 1.6–2.5 m from the gutter using the inducer lines, which is determined by the line works of the farmer.

**Fig. 1:**
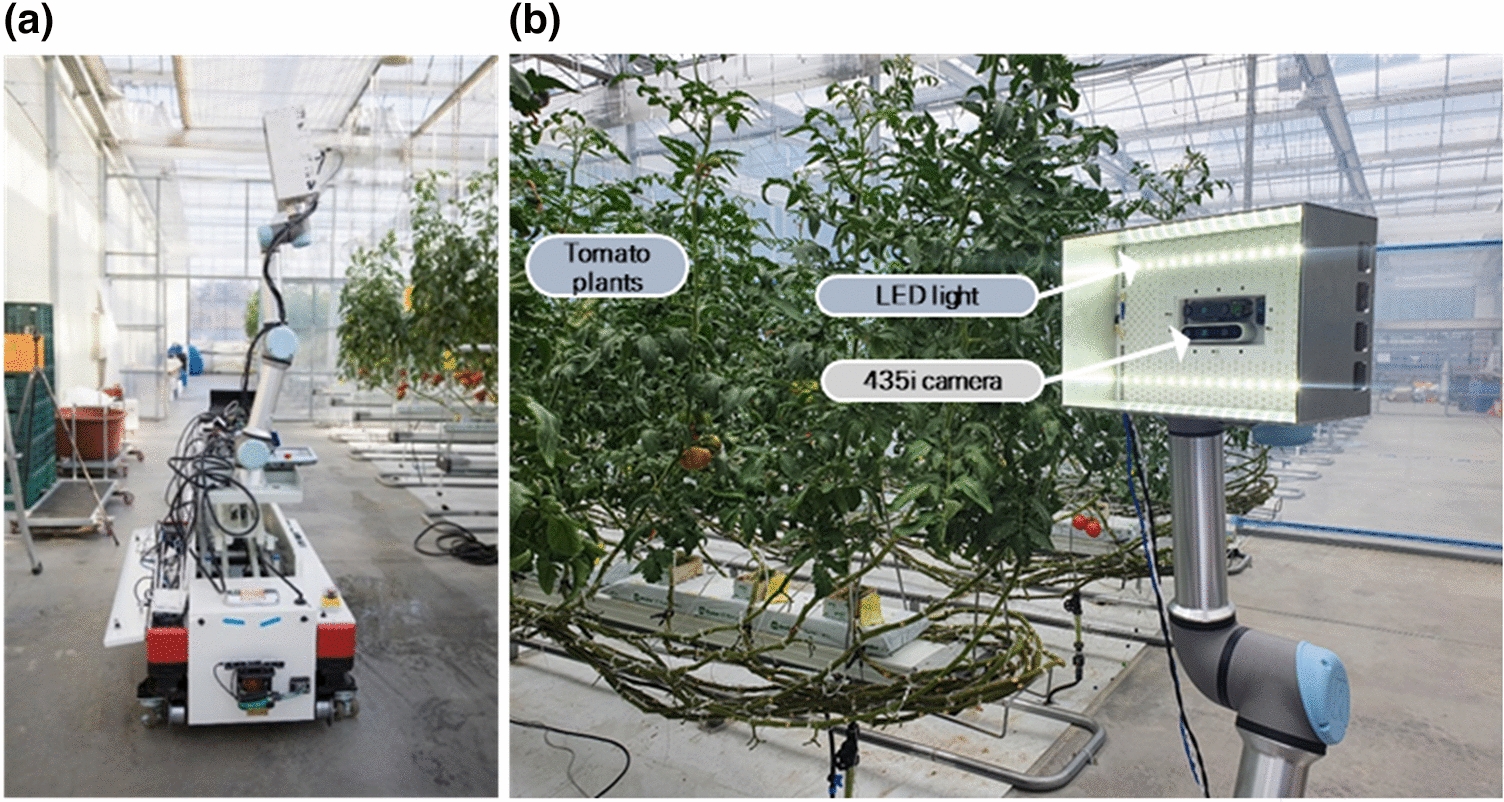
**a** Robot platform for image acquisition in greenhouse. **b** End effector for RGB depth image acquisition

To acquire the images, we used a vehicle placed on a robot platform capable of driving automatically in a greenhouse. A 5-joint UR5 (Universal Robots, Odense, Denmark) was used as a menu plater to fix the photographing unit at the position of the growing truss of the tomato plant. The menu plate operation was manually adjusted in the field, and the position of the photographing camera was kept constant at the center of the line. The image acquisition unit comprising a Realsense 435i camera (Intel, Santa Clara, CA, USA) acquired RGB and depth images. The maximum resolution of the camera was 1600 by 800. The measured images were collected on a mini-Windows PC (NUC, Intel, Santa Clara, CA, USA) and saved using a workflow developed in the Python programming language. Figure 1 shows a photograph of the robot platform and the measurement module used.

### CycleGAN implementation for segmentation of the tomato growing points

#### The CycleGAN structure

**Fig. 2:**
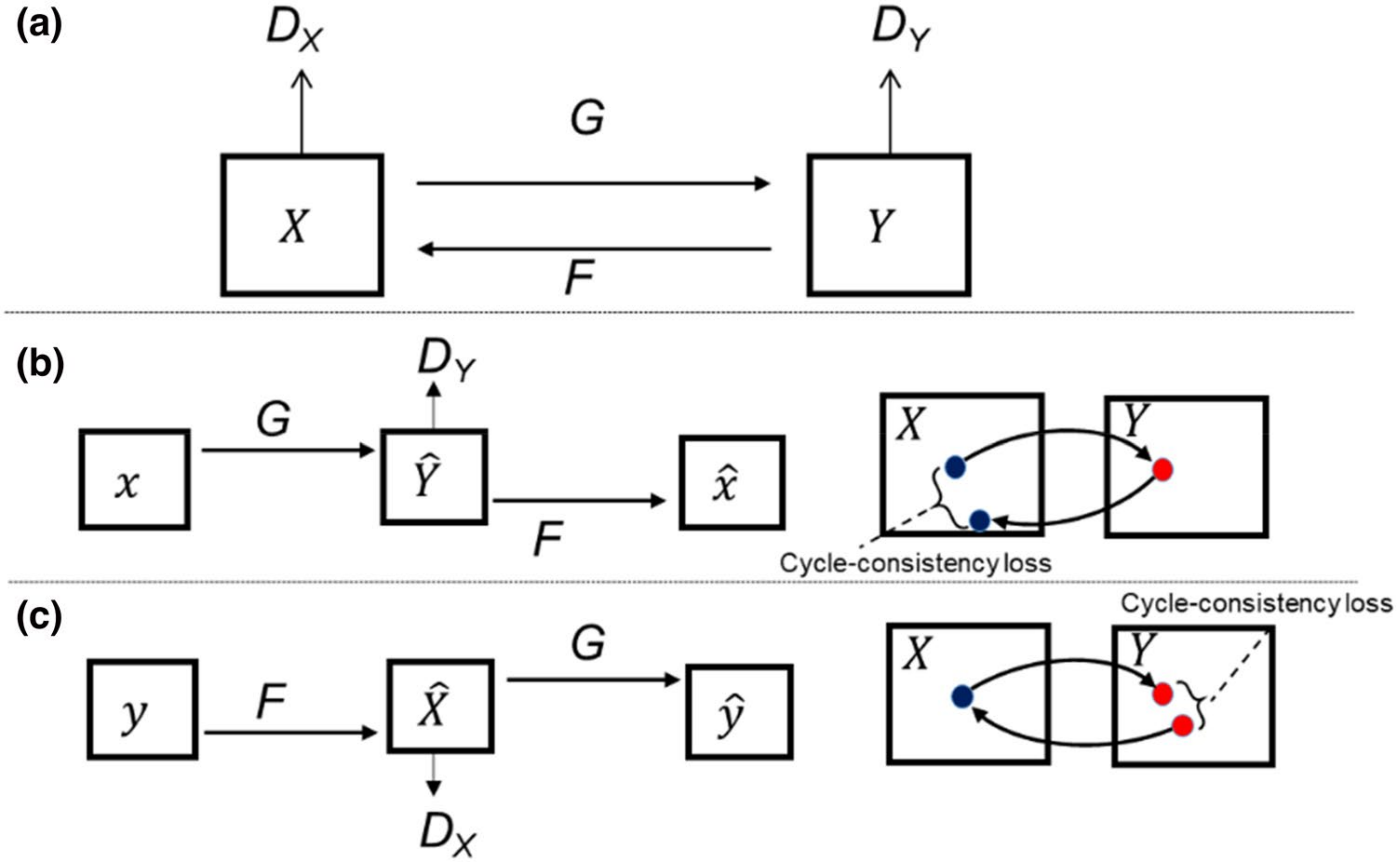
The CycleGAN structure. **a** Two mapping functions G: X → Y and F: Y → X. **b** Forward cycle-consistency loss. **c** Reverse cycle-consistency loss

The GAN is said to be successful when an adversarial loss makes the generated image indistinguishable from the actual photo. This loss is particularly powerful for image-creation tasks considering most computer graphics aim at achieving optimization [26]. The objective of the CycleGAN model is to learn the mapping functions between the two domains of X and Y using the given training samples ${\left\{{x}_{i}\right\}}_{i=1}^{N}$, where ${x}_{i}$ $\in$ X and ${\left\{{y}_{j}\right\}}_{j=1}^{M}$, where ${y}_{j}\in Y,$ which can be expressed as data distribution as $x \sim {p}_{data}(x)$ and $y \sim {p}_{data}\left(y\right).$ Zhu et al. [27] introduced two cycle-consistency losses (Fig. 2a), indicating that the starting position of $x$ must be reached when converting from one domain to another and vice versa. The forward cycle consistency loss is given as: x → G (x) → F (G (x))) ≈ x (Fig. 2b) and the reverse cycle consistency loss is given as y → F (y) → G (F (y)) ≈ y (Fig. 2c).

#### Application of CycleGAN for tomato depth image transaction

**Fig. 3:**
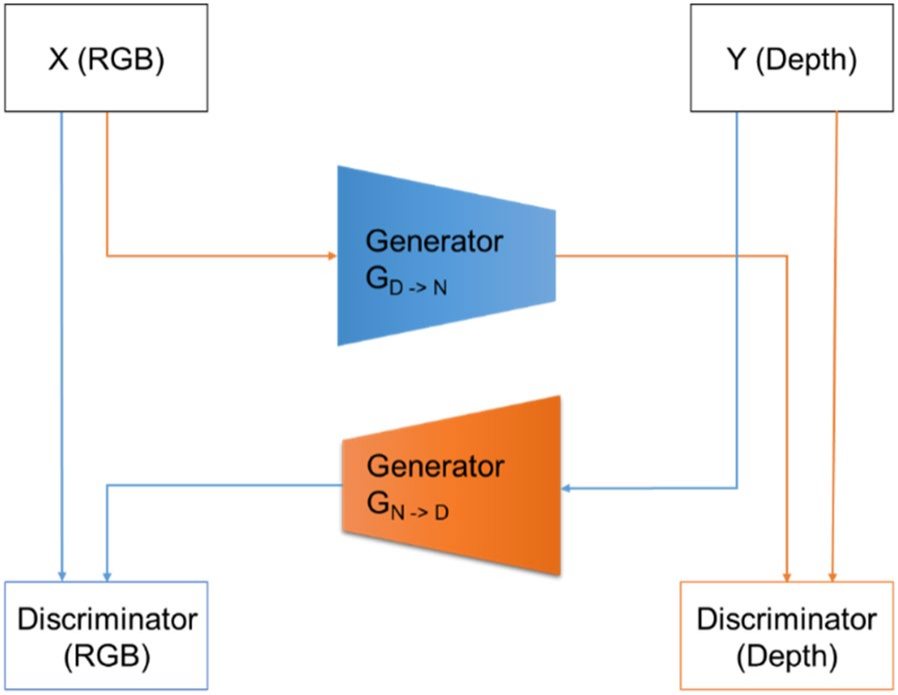
Schematic representation of the two cyclical generators of the CycleGAN

**Fig. 4:**
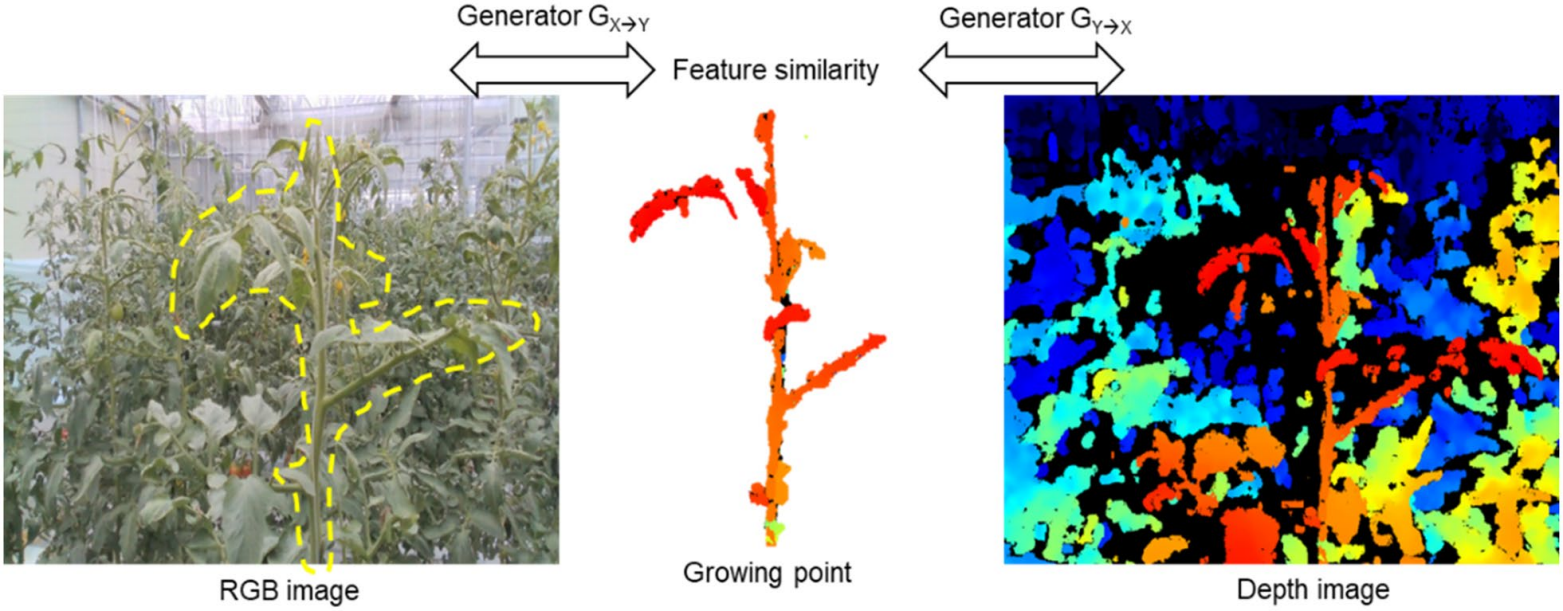
Relationship between the images generated from the X and Y generators and the image data to be extracted

As seen in Fig. 3, the RGB and depth images were obtained from the robot platform and the acquisition unit. As seen in Fig. 4 (left), a normal RGB image is similar to an image obtained from a normal camera. Figure 4 (right) shows an image with the depth technology applied, and the location information between the camera and the object in the video is displayed in a color table.

Using CycleGAN learning, we constructed a model that converts RGB images to depth images and vice versa, as seen in Fig. 4. The model was configured using approximately 356 sample images of the growing truss acquired from the image acquisition device at the fruit growing stage. Of the 356 sample images, 276 were used to train the CycleGAN model, and 80 samples were used for testing.

**Fig. 5:**
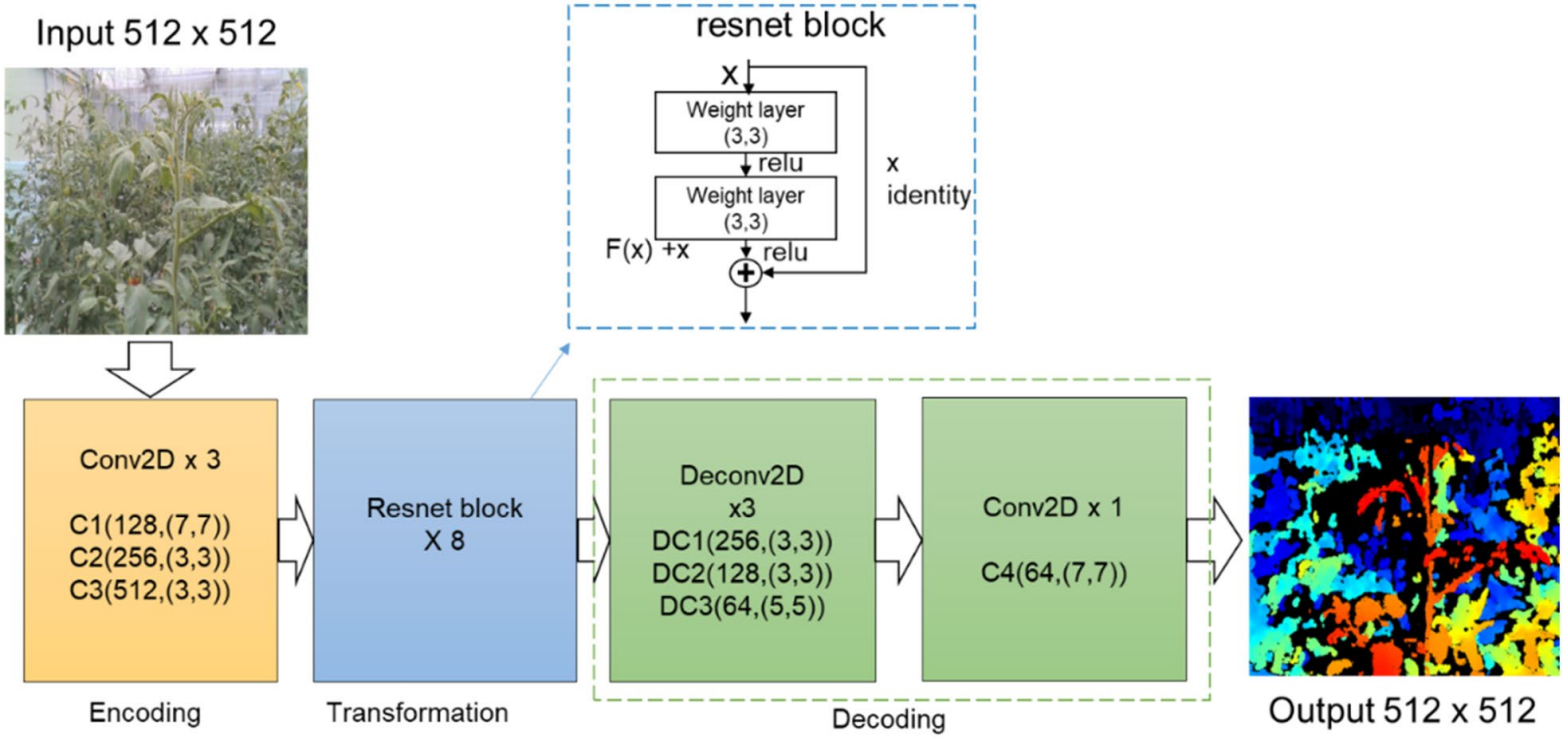
The CycleGAN generator architecture

Each CycleGAN generator comprises three sections: the encoder, transformer, and decoder. Figure 5 shows the components of each generator section. The 1600 × 900 pixels image used in this study was obtained as a raw value and resized to 512 × 512 pixels. First, the resized input image was fed directly to the encoder comprising three convolutional layers to increase the number of channels and decrease the representation size. The activated result was then passed to the transformer, which is a series of eight Resnet blocks that efficiently transfer information into the CNN structure. In the optimization problem, even if the number of layers is not deep, Resnet performs additional layer identity mapping by copying the learned layers; therefore, it can be used as a CNN structure verified in GAN image conversion technology [29]. The transformation result was then expanded by the decoder comprising two transpose convolutions that enlarges the representation size and one output layer, which then produced the final RGB image. Although each layer was followed by an instance normalization and Rectified Linear Unit (ReLU) activation function, it has become the default activation function for many types of neural networks. The basic form of ReLU is as shown in Eqn (1), and when it is differentiated, it can be expressed as Eqn (2). The rectified linear activation function overcomes the vanishing gradient problem, allowing models to learn faster and perform better.

1 $$f\left(\mathrm{x}\right)=\left\{\begin{array}{l}0 \, for\, x<0\\ x\, for\, x \ge 0\end{array}\right.$$

2 $${f}^{^{\prime}}(\mathrm{x})=\left\{\begin{array}{l}0\, for \,x<0\\ 1\, for x\, \ge 0\end{array}\right.$$

Furthermore, we built a discriminator that captures images and predicts whether they are real or fake. The real image is an actual RGB or depth image, and the fake image was generated by CycleGAN. The generator can be visualized in Fig. 5.

#### Image processing and evaluation methods for the extraction of growth points

The obtained depth image was subjected to pre-processing to extract the parts corresponding to the crop. First, a general RGB-based image of the crop showing the growing truss of the tomato plant was converted into a depth image. Because the depth image classifies the color of an object by distance, it can distinguish objects by finding boundaries (Fig. 4). Here, the growing truss of the closest part of the image we want has a relatively red color. Therefore, we extracted the area through hue, saturation, lightness (HSV). Although the extraction performance was better than the RGB-based method, the process was optimized with a trial-and-error method. In addition, morphological operations were performed to fill the remaining small fragments and the extracted area.

**Fig. 6:**
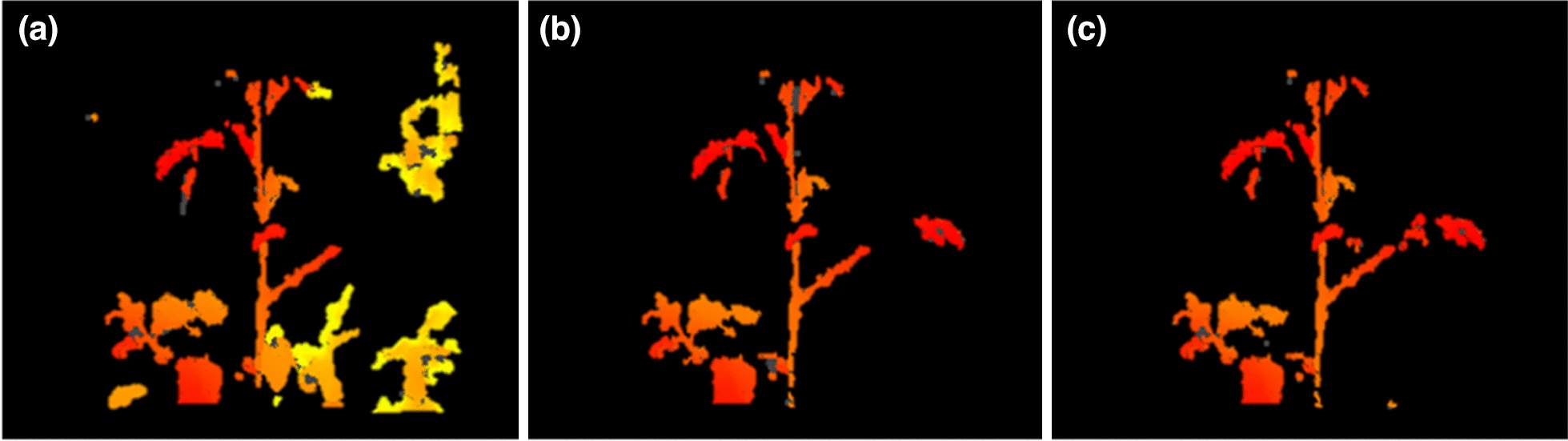
Comparison of crop extraction area using HSV range under three conditions: **a** H: 0 to 65 S: 150 to 255 V: 150 to 255, **b** H: 0 to 30 S: 180 to 245 V: 250 to 255, and **c** H: 0 to 30 S: 248 to 255 V: 240 to 255

We used the developed CycleGAN model in this study. The image was pre-processed by applying the HSV and Otsu thresholds. As seen in Fig. 6, the three HSV ranges were applied to the image preprocessing model in the development stage under the following three conditions: (a) H: 0 to 65, S: 150 to 255, V: 150 to 255, (b) H: 0 to 30, S: 180 to 245, V: 250 to 255, and (c) H: 0 to 30, S: 248 to 255, V: 240 to 255. As a result, the HSV range corresponding to (c), which was best able to identify the growing truss, was applied. The image designated by the HSV was then converted to be further binarized using the Otsu threshold.

**Fig. 7:**
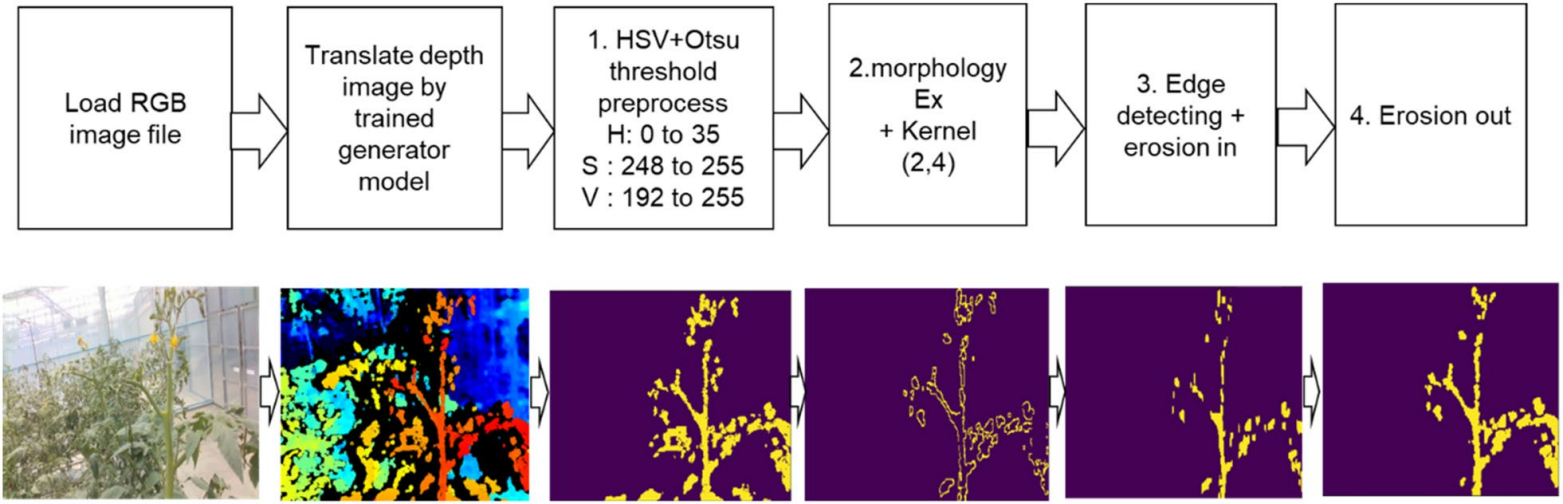
The entire image processing after CycleGAN conversion

The contour of the crop was determined using the morphology EX algorithm, which can perform advanced morphological transformations using basic erosion and dilation operations in place. In multichannel images, each channel is processed independently. The edge was detected from the contour obtained, and erosion was performed in one iteration using a 3 × 3 kernel to remove small objects corresponding to noise. Although this process can be applied universally in tomato greenhouses, it is difficult to use in general outdoor areas and places where the distance of the plantation from the camera is not fixed. The results of the entire image processing are shown in Fig. 7.

We compared the accuracy of the obtained growing truss image between the automated image processing technique and manual image extraction using 80 test samples. For the manual image extraction, a method of creating polygons and leaving ROI areas was intuitively determined by a person.

**Fig. 8:**
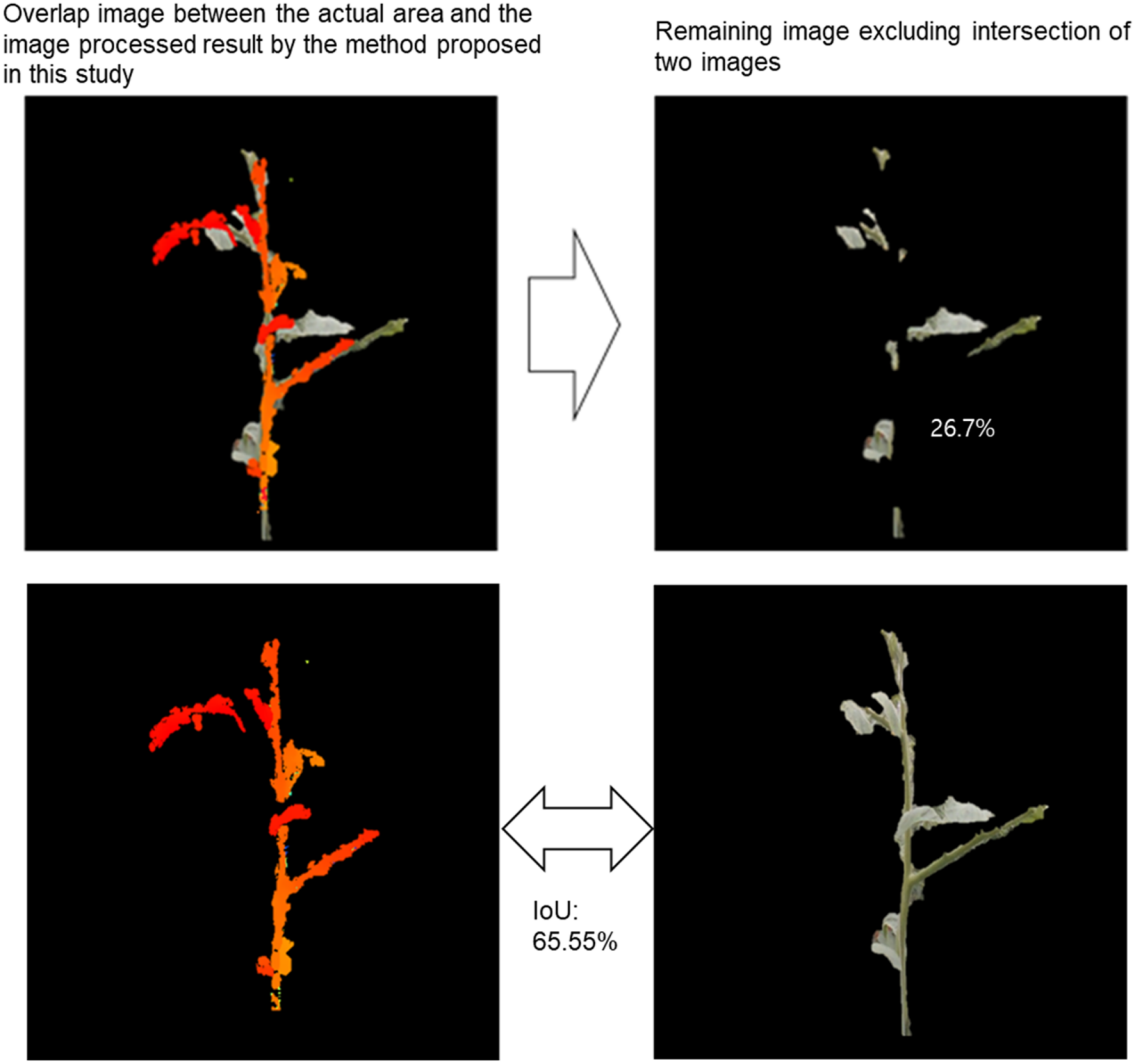
Ratio of the remaining image after removing the predicted pixel from the actual image, designated as false negative (FN), and when the actual image pixel is removed from the predicted image pixel, designated as false positive (FP)

The image extracted by manually was assumed to be the actual ROI. The extracted growing truss from the image processing technique and the actual ROI of the same size were overlapped, and the extracted image value at the same coordinate as the position of the actual growing truss was eliminated. The error rate was then calculated based on the number of pixels in the remaining images. Two indicators were calculated for the error rate: The residual ratio of the image after removing the predicted pixels from the actual image was designated as false negative (FN), and after removing the actual image pixel from the predicted image pixel was designated as false positive (FP). Equation (3) shows the calculation for the residual ratio. In addition, as a standard segmentation method, mean intersection over union (mIoU) was calculated for evaluating the image segmentation methods. Figure 8 shows the specific calculation method for FN and mIoU using the resulting image and eqn (4) shows the details of mIoU.

3 $$\mathrm{Residual\, ratio }\,(\%)=\frac{The \,number \,of \,pixels\, in\, the\, remaining \,image}{Total\, number\, of\, pixels\, in\, the\, ROI\, of\, crop } \times 100$$

4 $$\mathrm{Mean \,intersection\, over union }(\mathrm{mIoU})\, (\%)=\frac{Area \,of \,overlap}{Area\, of\, union } \times 100$$

A receiver operating characteristic (ROC) curve shows the performance of the segmentation model in units of pixels of the image. The ROC curve compares the true- and false-positive rates, and shows that the wider the ratio of the true positive rate, the better the classification model.

## Continuous measurement of images of robots for field applicability of CycleGAN

**Fig. 9:**
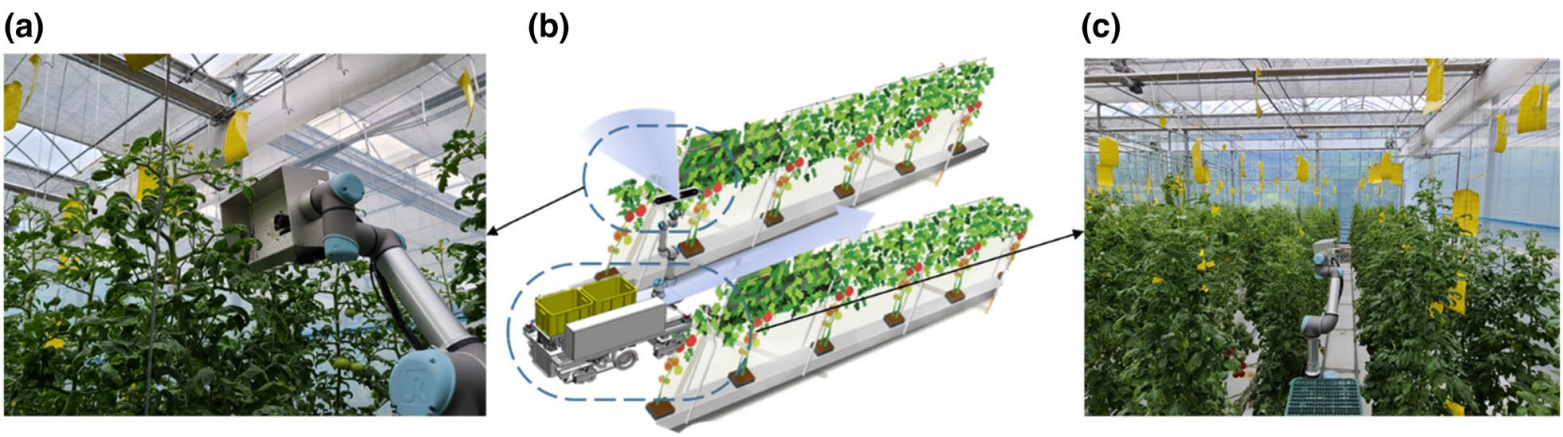
Schematic representation of the experiment for applying the developed algorithm to sequentially captured and registered RGB images. **a** Front view of the scanning area of the robot. **b** Experimental schematic. **c** Actual robot running direction

We conducted a field applicability test to examine the possibility of measuring the desired tomato stem section in the greenhouse crop bed driving. The vehicle was driven between the planting spaces in the greenhouse in a straight line and continuously scanned images of a particular location. We only collected the RGB images from the RealSense camera, which were then converted using the previously developed CycleGAN. Further, an image processing technique was applied to extract the ROI from the image. The RGB images were captured continuously at intervals of 1 min by advancing approximately 5 m for every 2 s by fixing the forward speed of the robot to 0.5 m/s. We simultaneously performed the image conversion and extraction of the ROI on the stem. Figure 9 shows the performance of the growth measurement experiment inside an actual greenhouse.

## Results

### Training results of the CycleGAN

**Fig. 10:**
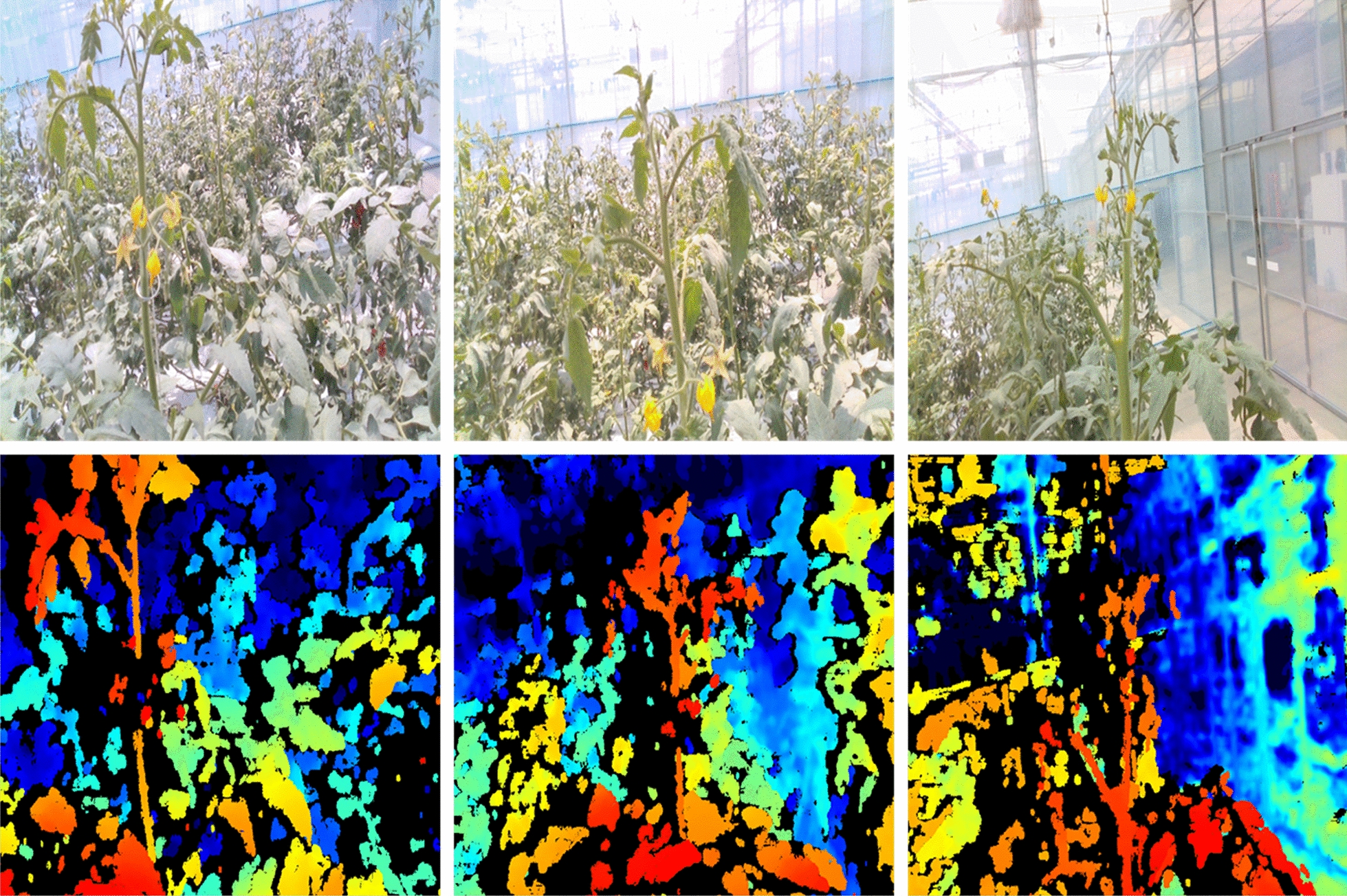
RGB and depth image to be used in the acquired data set (top: RGB, bottom: depth)

The growing truss of the tomato plant was collected through the camera attached to the vehicle-based robot arm proposed above. A total of 350 pairs of images were collected and CycleGAN learning was performed. This data can be found in the Additional file 1 provided. Figure 10 shows the collected data, the shape of the growing tomato truss, and the greenhouse cultivation environment.

**Fig. 11:**
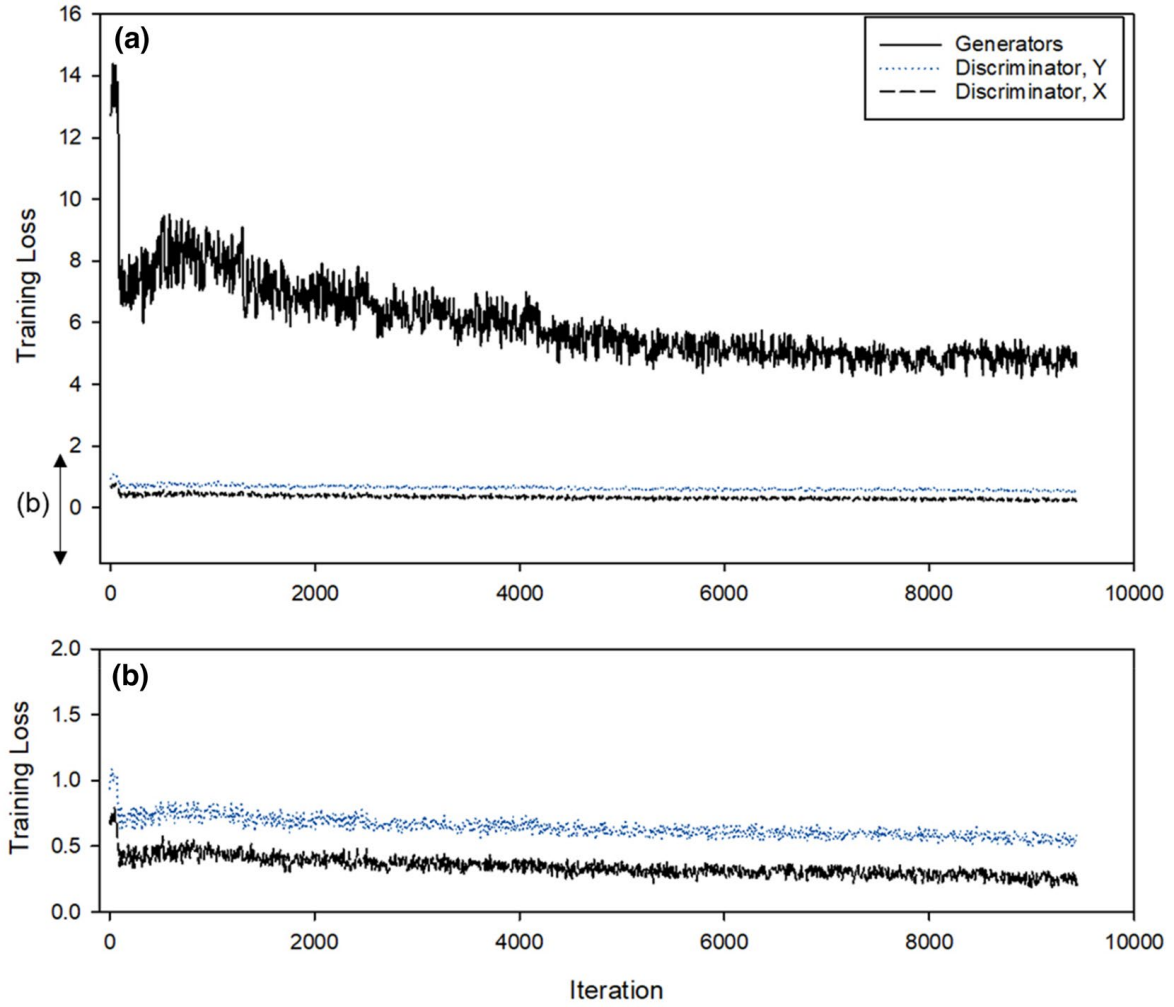
Changes to the overall loss. **a** Change in loss of discriminator X and Y. **b** On training the proposed CycleGAN structure

The CycleGAN was trained for approximately 9600 iterations in five batches using approximately 276 training samples. At this time, the changes in the loss of the generator and discriminators X (Dx) and discriminators Y (Dy) can be confirmed, as seen in Fig. 11. First, the generator loss was observed to have converged in the half at a certain level, although there was some loss in the beginning. The discriminator gradually converged to 0.5 for Dx, but further converged to approximately 0.55 for Dy. For depth to RGB generation, an error with the actual sample was confirmed. However, the learning performance, which was mainly used in this study, seemed to have been secured in the RGB to depth image to an extent.

**Fig. 12:**
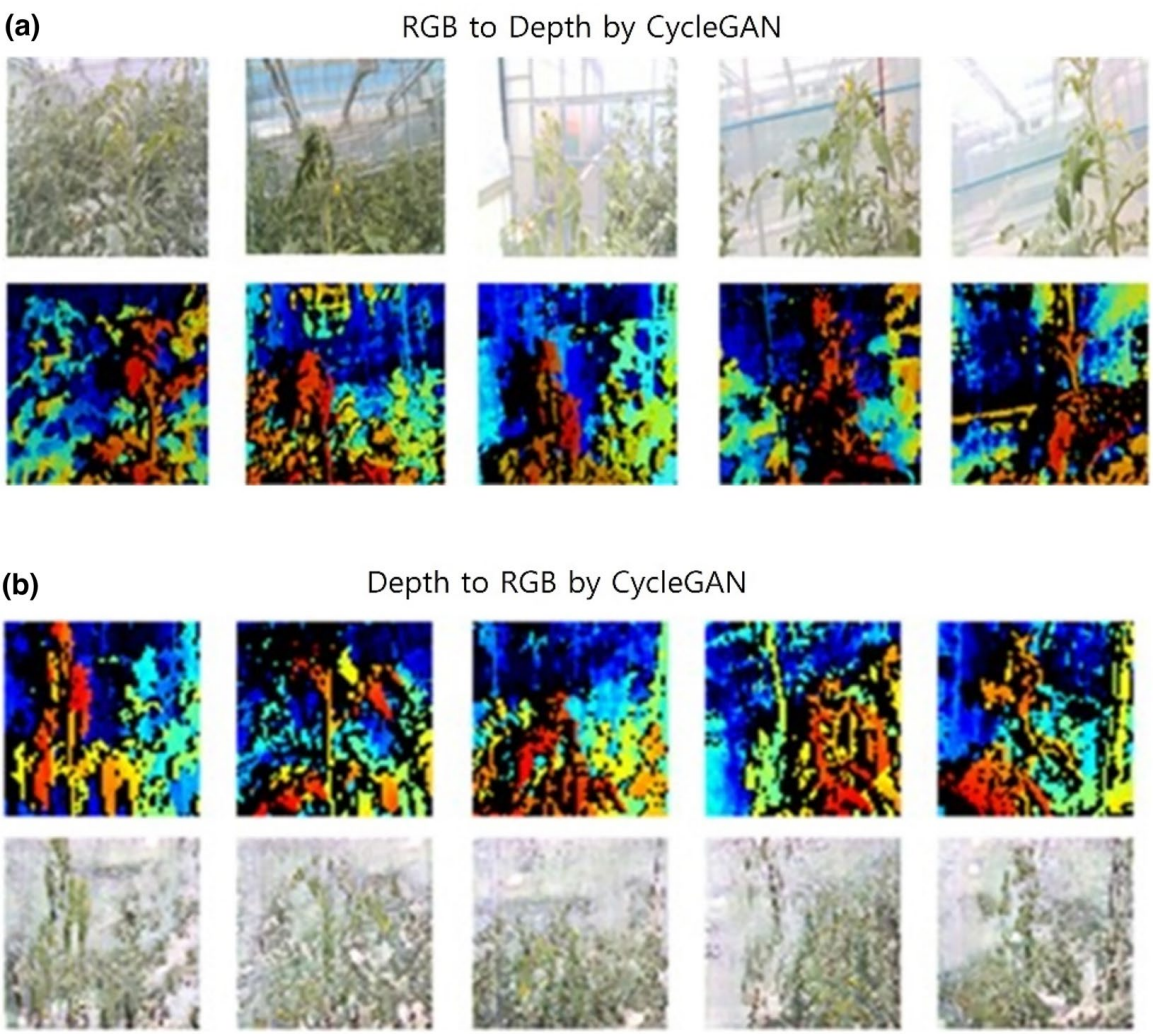
Results of RGB to depth image conversions (**a**) and realizing the depth in RGB (**b**) through CycleGAN’s 8800 iteration learning

Figure 12 (a) shows the RGB-to-depth learning process. It was confirmed that the generator results obtained at 8800 iterations clearly depicted the appearance of crops as compared to initial iterations in the initial learning period. In addition, the RGB color differed based on the size and shape of the crop, and a similar pattern was observed in the depth images. Conversely, for the depth-to-RGB image, a low-quality crop image was obtained considering the input image could not generate high-quality images, as seen in Fig. 12(b). Although the appearance, characteristics, and color of the crops were simulated like real RGB images, it was difficult to distinguish specific features with the naked eye.

### Accuracy of image extraction

**Fig. 13:**
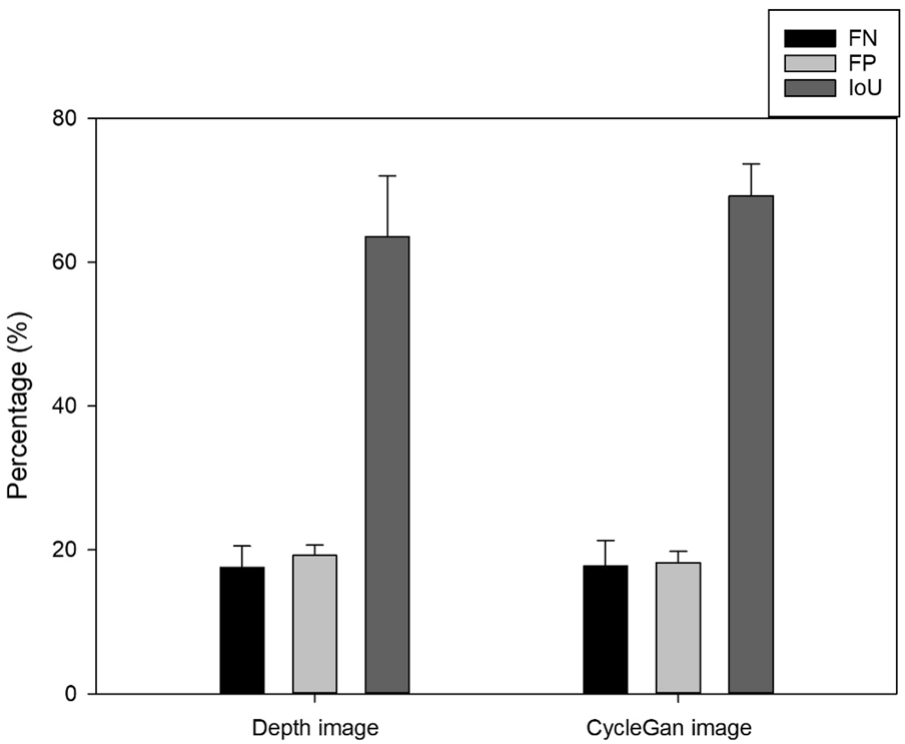
Comparison between the depth and CycleGAN image of a manually specified ROI using the proposed image processing technique, and the FP, FN, and mIoU values in pixel units

**Fig. 14:**
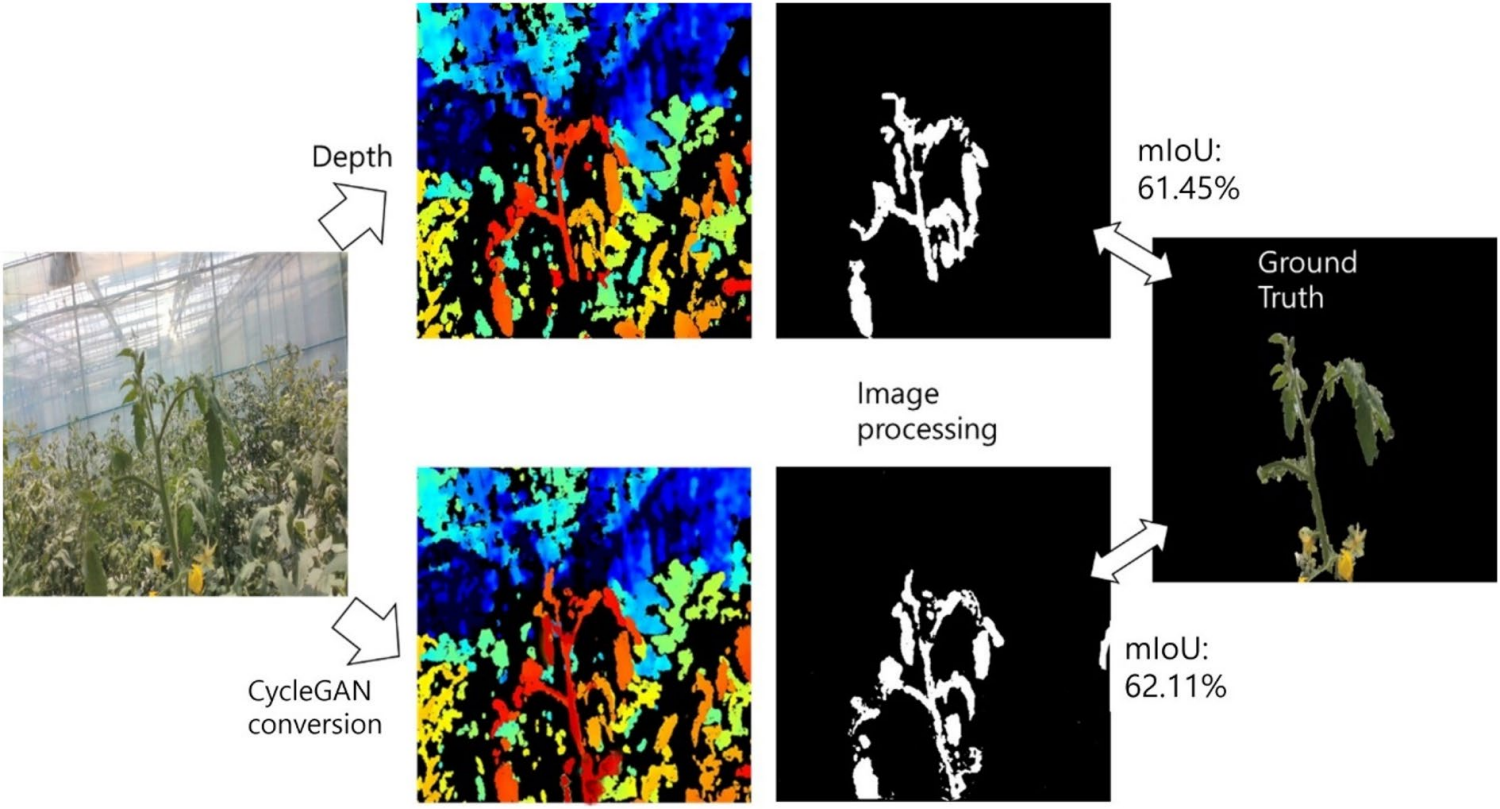
Comparison of the CycleGAN and depth image with the actual extracted area and cross-segmentation values through mIoU

The conversion from an RGB image to a depth image was mainly for the segmentation of target crops, and we verified the accuracy of FN and FP as an evaluation method. From the previously developed CycleGAN models, the results were inferred using 8800 iterations, whereas the image pre-processing and growing truss extraction image processing methods were the same. We obtained results as seen in Fig. 13 by comparing the results based on 80 images that were not used for model training. When the FN and FP were calculated using the image obtained from the depth camera, we obtained an approximate value of 17.55% ± 3.01% and 17.76 ± 3.55%, respectively. Similarly, on converting the image using CycleGAN, the FN and FP were approximately 19.24% ± 1.45% and 18.24% ± 1.54%, respectively. In terms of error probability, the CycleGAN and depth images were compared with the actual extracted region and crossed segmentation values through mIoU as shown in Fig. 14. Among the total test samples, when segmentation was performed using the depth image, the mIoU was 63.56 ± 8.44%; whereas, when segmentation was performed through CycleGAN, the mIoU was 69.25 ± 4.42%. Additional analysis result samples for each algorithm are presented in the attached mIoU sample file.

**Fig. 15:**
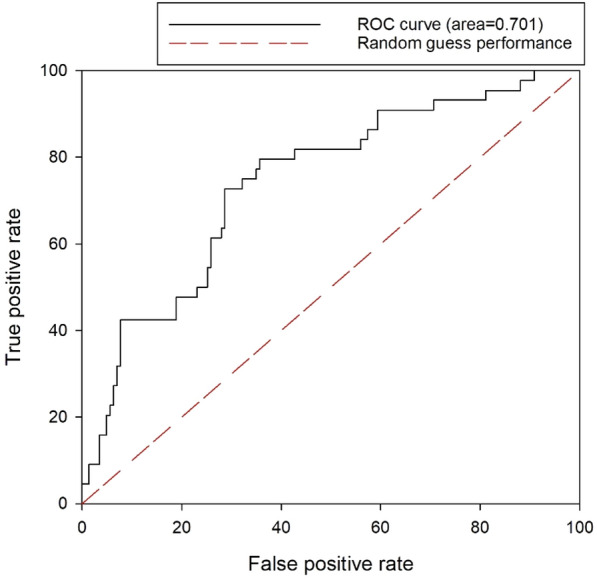
ROC curve for evaluation of segmentation using CycleGAN model

Figure 15 shows the results of the ROC curve for the evaluation of segmentation using the CycleGAN model. The red dotted line in the figure corresponds to the performance by random guessing. Based on this, the more the curve is biased in the true-positive rate region, the better the model performance can be estimated, and this area is calculated as the area under curve (AUC). In this CycleGAN model, when segmentation of the test set was applied, an AUC of 0.701 was obtained.

### Field application results for continuous detection

**Fig. 16:**
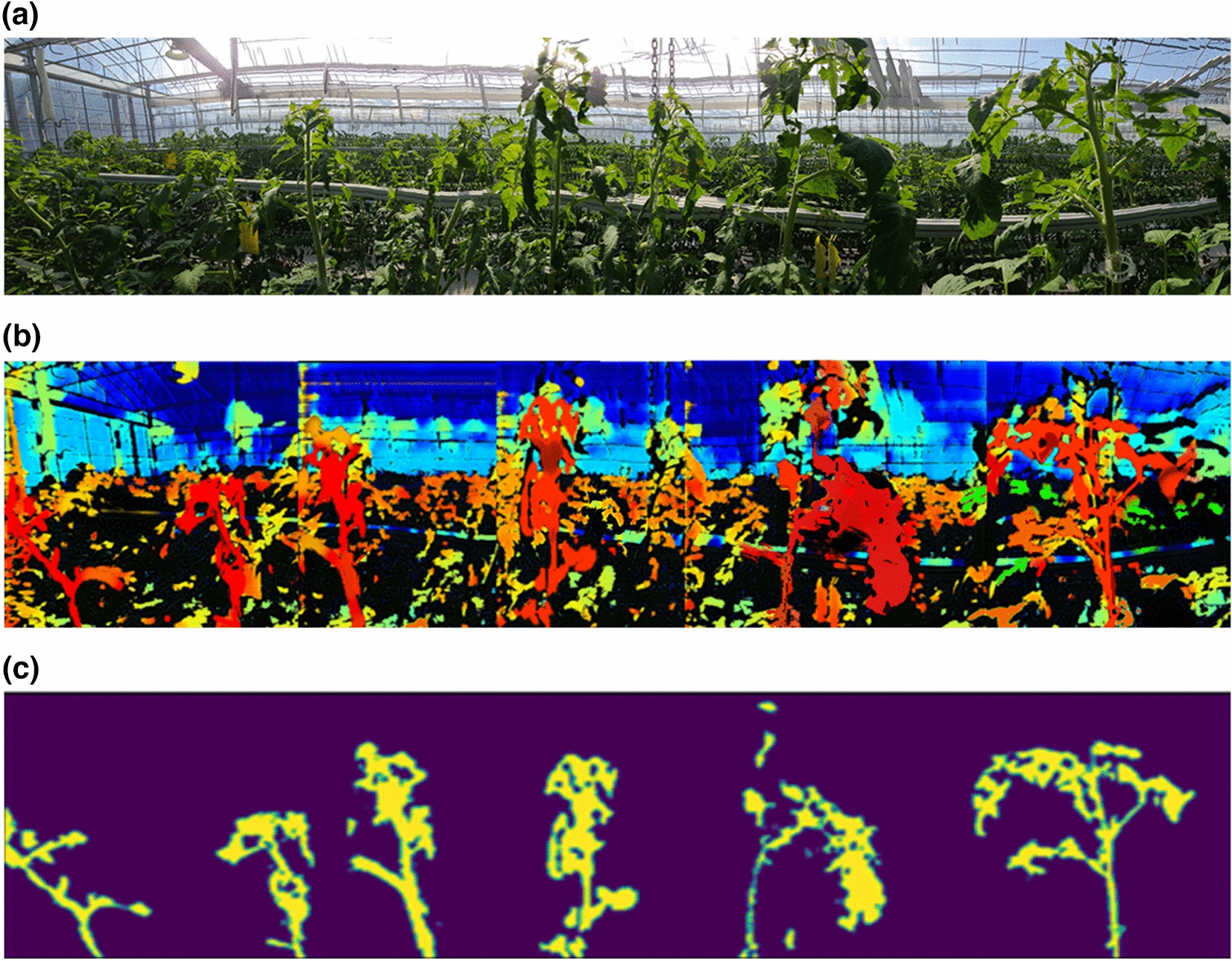
**a** RGB image acquired using on-site robot platform. **b** Depth image created using CycleGAN. **c** View underneath the growth point location extracted through image processing

Because this study aims to extract the growing truss of tomato crops in a greenhouse using CycleGAN and image processing technology, the possibility was confirmed by field application experiments. Figure 16a shows the result of continuously acquiring and matching images with a height of approximately 3.5 m, wherein the growing truss can be confirmed while the image acquisition vehicle advances inside the greenhouse. After advancing for 5 m, it was confirmed that approximately six crops were unevenly distributed. Figure 16b shows the result of converting the image into a depth image using the developed CycleGAN model. Similar to the actual depth image, the image showed the object to be segmented. The depth in the image was indicated in red for the closer crops, and in blue for the farther crops. Finally, the result of extracting the growing truss, i.e., the stem and leaves of the tomato plant, using the image processing technique, can be confirmed from Fig. 16c.

## Discussion

Past researches have focused on applying the vision of crops in fruit-oriented research. However, in the case of stem plants, such as tomatoes, the state of the growing truss, which grows continuously, can be used as an important indicator to determine the future yield. Therefore, we conducted research on image processing techniques to identify the growing truss of the tomato plant and used deep learning to acquire highly efficient results. We first devised an image processing technique to segment the part of the plant that could be identified as a growing truss through CycleGAN using a depth image and a simple RGB image converted into a depth image. Given that the CycleGAN is useful in image conversion, it was useful for recognizing objects that existed in two images, which successfully extracted the growing truss. Furthermore, it was possible to convert the color of the growing truss to the color of the depth, which was red in the prepared training set. Owing to the CycleGAN method, both transformations can be applied, which has already been proven in a previous study [23, 24]. If we compare the purpose and approach of the existing segmentation studies on tomato images [11, 12], it can be seen that many studies have focused on the analysis of tomato fruit, whereas we focused on the growing truss (the stems and leaves). Although, it is very difficult to classify the stems and leaves of the tomato plant because the growing environment is very dense, the possibility of an approach using depth imaging was confirmed in this study.

**Fig. 17:**
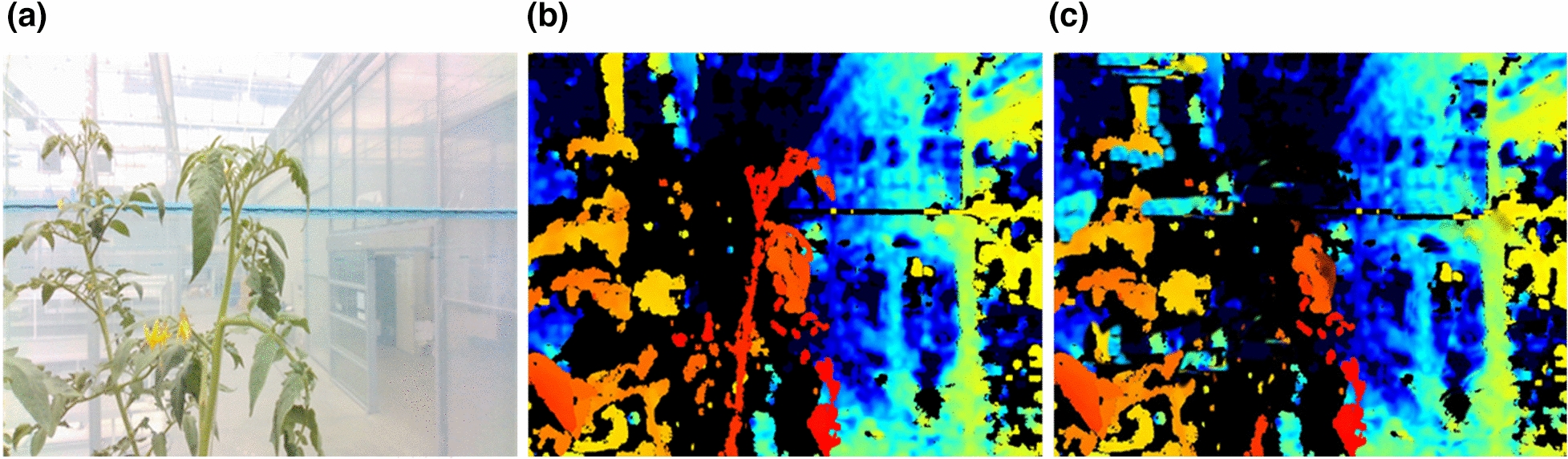
Failed cases due to blurred focus of the depth camera. **a** RGB image. **b** Growing point in focus. **c** Focused farther from the growing point

Although the identification error rate was lower when using the depth image, as seen in Fig. 13, the average error rate was less than 20% in the two techniques, which indicated that the segmented object was not another ROI. The difference in error rate between the two methods was 2–3%, and it was determined that this was not a clear performance difference. The reason was that the ground truth was manually referred to because the error rate of this ground truth itself exists. However, in standard deviation, CycleGAN confirmed the result with a minor deviation value, which could have been due to the depth camera image being applied to the field considering the tendency of the camera to lose focus at proximity with a 10% probability, as seen in Fig. 16c. However, because this was related to the applicability field of the camera, it was not considered in this study. Objects that remain unrecognized by the depth camera are termed as a failure case, as seen in Fig. 17, can cause problems in field applications in the future. However, this problem did not occur in the depth image converted using CycleGAN, considering it was already being used in the training set stage.

Additionally, it is often necessary to prepare annotated image samples as training data for artificial intelligence algorithms that recognize objects by judging through human intellectual contribution, and a large amount of data samples is required to verify the accurate performance. However, the results of CycleGAN and the image processing method proposed in this study have confirmed that preparing the annotated image samples is not required.

In the future, robots will be required in agriculture to automatically measure plant growth. However, the robot must accurately recognize the growing truss to establish a menu plating strategy to choose the desired growing truss. In this study, we adopted CycleGAN, an artificial intelligence image conversion technique, as the first step for the robot to recognize the growing truss. Consequently, the robot was able to effectively extract the growing truss using the matched image even in field applications. In the field applicability verification experiment, the moving robot matched several images and finally converted the image using CycleGAN. The result was verified by only extracting the growing truss from the image. However, an irregular connection of images was observed during the registration, and the CycleGAN structure used when converting to depth applies to only 512 × 512 images, making a grid shape inside the images. As this applies to all images using deep learning, it is necessary to solve the problem using an algorithm for the flexible application of the input layer structure. Nevertheless, the result indicated that the application of unmanned robots in agriculture in the future has been well considered.

In future research, we will consider using a method of acquiring optimal images by menu plating the robot arm once the growing truss is recognized. In addition, the result of converting the depth image to an RGB image, although not addressed in this paper, is worthy of discussion as a future study (Fig. 12). In conclusion, remote-operated and unmanned machines have a high potential for use in the agriculture industry [30], and creative results can be achieved when fused with artificial intelligence.

## Conclusions

In this study, we developed a technique for extracting the growing truss in tomato plants in a greenhouse using image processing techniques based on the image information obtained by a robot platform and images of the growing truss captured by a depth camera. Furthermore, a study was conducted to convert the characteristics of two images, that is, converting RGB images into depth images, using the CycleGAN algorithm. Discriminators X and Y used in the loss of learning process converged to 0.43 and 0.65, respectively. The image information converted using CycleGAN was further used to compare the performance of the extraction of growing truss. The FN and FP values based on the images from the depth camera were approximately 17.55% ± 3.01% and 17.76 ± 3.55%, respectively. Similarly, using CycleGAN, the FN and FP values were approximately 19.24% ± 1.45% and 18.24% ± 1.54%, respectively. When using depth image, the mIoU was 63.56 ± 8.44%, and when segmentation was performed through CycleGAN, the mIoU was 69.25 ± 4.42%. In terms of error probability, CycleGAN exhibited a higher value. Finally, we performed field application tests to determine the growing truss of tomatoes, wherein the continuously scanned image information was converted into depth images using CycleGAN. In the future, the proposed approach is expected to be used in vision technology to scan the tomato growth indicators in greenhouses using an unmanned robot platform.

## Supplementary Information

**Additional file 1.** Depth and RGB pair images of tomato growing truss and developed CycleGAN model.

## Data Availability

The datasets supporting the conclusions of this article are included within the article (and its Additional file 1).

## Abbreviation

GAN: Generative adversarial networks
